# Wet-tip versus dry-tip regimes of osmotically driven fluid flow

**DOI:** 10.1038/s41598-019-40853-7

**Published:** 2019-03-14

**Authors:** Oleksandr Ostrenko, Jochen Hampe, Lutz Brusch

**Affiliations:** 1 Centre for Information Services and High Performance Computing, Technische Universität Dresden, 01062 Dresden, Germany; 2 MOSAIC Group, Center for Systems Biology Dresden, 01307 Dresden, Germany; 3 Medical Department 1, Universitätsklinikum Dresden, 01307 Dresden, Germany

## Abstract

The secretion of osmolytes into a lumen and thereby caused osmotic water inflow can drive fluid flows in organs without a mechanical pump. Such fluids include saliva, sweat, pancreatic juice and bile. The effects of elevated fluid pressure and the associated mechanical limitations of organ function remain largely unknown since fluid pressure is difficult to measure inside tiny secretory channels *in vivo*. We consider the pressure profile of the coupled osmolyte-flow problem in a secretory channel with a closed tip and an open outlet. Importantly, the entire lateral boundary acts as a dynamic fluid source, the strength of which self-organizes through feedback from the emergent pressure solution itself. We derive analytical solutions and compare them to numerical simulations of the problem in three-dimensional space. The theoretical results reveal a phase boundary in a four-dimensional parameter space separating the commonly considered regime with steady flow all along the channel, here termed “wet-tip” regime, from a “dry-tip” regime suffering ceased flow downstream from the closed tip. We propose a relation between the predicted phase boundary and the onset of cholestasis, a pathological liver condition with reduced bile outflow. The phase boundary also sets an intrinsic length scale for the channel which could act as a length sensor during organ growth.

## Introduction

**Figure 1:**
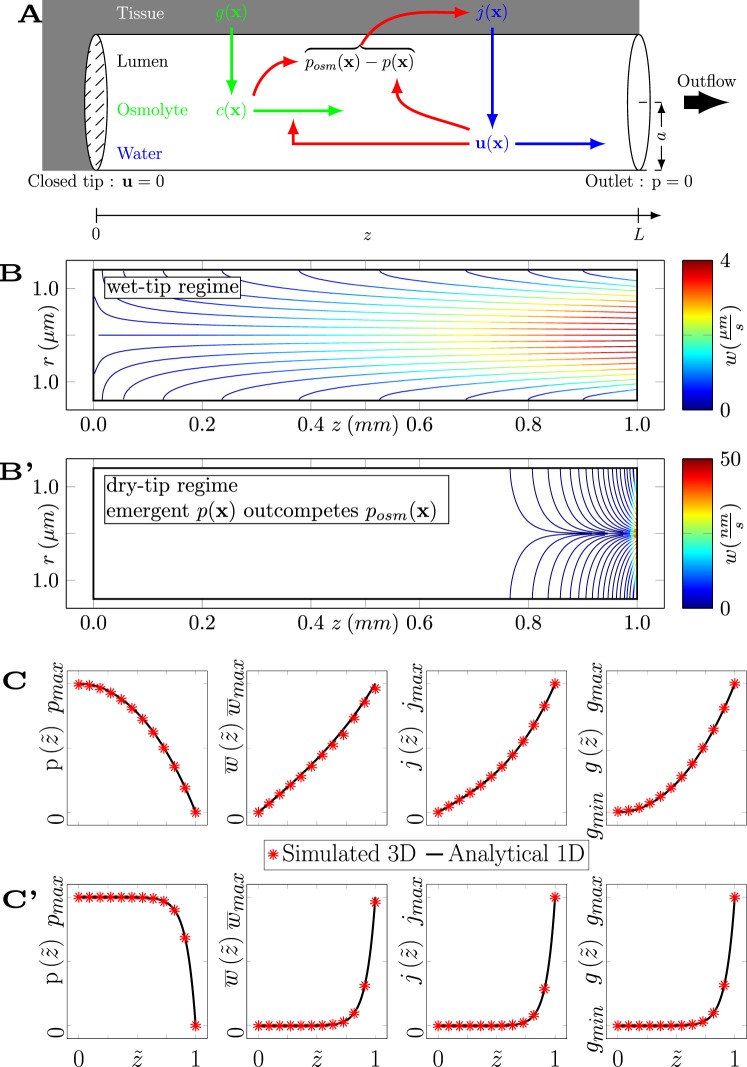
Model and solutions of osmotically driven fluid flow. (**A**) Model of a secretory system (cylinder with radius *a*, length *L* in cylinder coordinates with *z* along the centerline of the channel) with osmolyte (green) and water (blue) transport coupled through mechanical feedback loops (red arrows). Water influx *j*(**x**) through the lateral surface is driven by local osmotic pressure *p*_*osm*_(**x**) from osmolyte concentration *c*(**x**) and opposed by hydrostatic fluid pressure *p*(**x**), therewith forming one negative feedback loop (upper red arrows). Washout of osmolytes (*c*(**x**)) by flow (**u**(**x**)) constitutes a second negative feedback loop (lower red arrow). Boundary conditions for flow are mixed: zero velocity at the closed tip ($z=0$) and ambient pressure (set to zero) at the open outlet ($z=L$), hence a pressure gradient between the boundaries is not imposed but self-organizes. (**B**,**B’**) Typical results of 3D flow simulations depicted as streamlines on central plane for parameters $c=const$ and $M={M}_{0}$ (**B**,**C**), $M=100\,{M}_{0}$ (**B’**,**C’**) as given in Tables 1 and 2, color denotes velocity. Two qualitatively different flow regimes, a wet-tip regime with flow all along the channel and a dry-tip regime with ceased flow near the closed tip of the channel, are encountered. Note the beginning of streamlines at the channel surface, see Methods for details. (**C**,**C’**) Analytical solutions for pressure $p(\tilde{z})$ (Eq. 12), average axial velocity $\overline{w}(\tilde{z})$ (Eq. 27), osmotic water influx $j(\tilde{z})$ (Eq. 31) and osmolyte secretion $g(\tilde{z})$ (Eq. 29) profiles compared to results of numerical simulations as in (**B**,**B’**).

A variety of fluid transport processes such as phloem flow, primary saliva, sweat, pancreatic juice and bile production are not propelled by an externally applied pressure difference along the flow path but rely on osmotic driving due to components in the fluid called osmolytes^1–3^. The range of osmolytes spans from small ionic compounds like sodium, potassium and chloride ions up to complex compounds like sugars and bile salts. The resulting excess osmotic pressure between the lumen and the surrounding cells is the driving force of water flux across cell membranes and epithelial cell layers^4,5^. Historically, the osmotically driven fluid flow mechanism was first studied in plants by Ernst Münch as “Druckstromtheorie”^6^. Since the osmolytes that are setting the fluid in motion are themselves advected with the fluid and the fluid pressure counteracts the osmotic pressure, the transport theory has to consider the *bi-directional coupling* between the spatio-temporally varying osmolyte concentration profile and the hydrodynamic problem, see red arrows in Fig. 1A.

Most secretory organs encompass an intricate network of channels^7^. However, to focus on the effects of the regulatory feedback, the geometry of the organ’s lumen has typically been abstracted, neglecting branch points, as an elongated channel with a closed tip and an open outlet. Correspondingly, the transport theory encompasses a *mixed* set of boundary conditions, zero flow velocity at the closed tip and a fixed pressure at the open outlet. Moreover, the entire lateral boundary acts as a fluid source, the strength of which is determined by feedback from the emergent pressure solution itself. This is in marked difference to many other flow systems including engineering applications with a given pressure difference between channel boundaries. For a fully coupled osmolyte-flow system with distributed osmolyte sources and water sources, therefore, (i) the system behavior and its transport capacity as a function of parameters as well as (ii) functional limitations due to elevated fluid pressure are not fully understood yet. Another open question is whether dynamic regulation of channel geometry and/or fluid properties can assure outflow from the channel’s closed tip. Moreover, since fluid pressure is difficult to measure directly inside tiny secretory channels *in vivo*, a deeper theoretical understanding of the coupled osmolyte-flow problem will be useful.

Osmotically driven fluid transport has been studied in a series of mathematical models sprouting from the pioneering work by Kedem and Katchalsky^8^ into describing simple cyst expansion^5,9–11^, phloem flow in plants^12–20^ and fluid secretion in mammalian glands^4,21–23^. Recently, Rademaker *et al*. used their phloem flow model to establish a biomechanical length limit for plant needles around the observed 5 *cm*^20^. Among these theoretical studies, some considered the bi-directionally coupled osmolyte-flow system but had to apply simplifying assumptions for solving the model, i.e., either negligible hydrodynamic pressure compared to the osmotic pressure, thereby uncoupling the problem^21–23^, or considering a limit case of small solute secretion rate^4^ and, additionally, diffusion dominating over advection^19^, or replacing the mixed set of boundary conditions by pure velocity boundary conditions and not solving for the pressure profile explicitly^12–18^.

In this work we explore the effects of the mechanical feedback loops, see Fig. 1A, by taking a combined analytical and numerical approach to the bi-directionally coupled system and evaluate its implications for bile secretion in the liver. In the following sections we derive the model equations for the distributed secretion-advection problem of osmotically driven fluid flow, then map the model equations at steady-state to a single ordinary differential equation (ODE) of Abel type and obtain relevant particular solutions which predict the fluid pressure profile as a function of geometrical and material parameters. When parameter values are changed, the analytic solution reveals a transition between continuous flow from the closed tip on versus stalled flow with osmolyte accumulation near the closed tip, which we will term “wet”-tip and “dry”-tip parameter regimes, respectively. These analytical results are confirmed and complemented by numerical simulations of the full model in three-dimensional space. We finally apply the generic model to distributed bile secretion and flow in the liver and propose a relation to biliary pathology.

## Results

### Mathematical model of osmotic fluid secretion and flow

We consider the flow of a dilute solution of an osmotically active solute, approximated as an incompressible Newtonian fluid of uniform viscosity, that is driven by osmotic water sources distributed along the lateral surface of a cylindrical channel, see Fig. 1A. The lateral surface of the cylinder with spatially uniform and temporally constant radius *a* and length $L\gg a$ is not only covered with water pores but also with active transporters for osmolytes with flux density *g*. For the example of bile canaliculi in the liver, the lateral cylinder surface is constituted by the apical membrane of many hepatocytes^7^. Tight junctions seal the gaps between the apical membranes of neighboring hepatocytes. Energy-consuming transmembrane pumps, including BSEP, MDR2, MRP2, actively transport osmolytes even against any osmolyte concentration gradient across the apical membrane^24^. We regard any process in the surrounding tissue at steady state and factored into the osmolyte secretion flux density *g* such that *g* becomes a fixed parameter, independent of osmolyte concentrations in lumen and surrounding cells.

A cylindrical coordinate system ${\bf{x}}=(r,\varphi ,z)$ is introduced and the *z*-axis is aligned along the centerline of the channel with its origin at the closed tip. This closed tip represents the dead end of secretory channels like the tips of bile canaliculi in the pericentral area of a liver lobule where no direct contact exists between the channel and any circulatory system^7^. Moreover, for the cleft system spanned by the lateral membranes of epithelial cells, this closed tip corresponds to a sealed tight junction preventing any leakage^23^. Hence, the closed tip ($z=0$) is modeled as a rigid non-permeable wall while the other end ($z=L$) is an open outlet, representing e.g. a large bile duct or a reservoir.

This model system can be studied by three-dimensional computational fluid dynamics simulations of the Navier-Stokes equations, see Fig. 1B,B’ and Methods. A typical resulting flow pattern is illustrated in Fig. 1B with streamlines on the center plane of the computed three-dimensional velocity field colored by the magnitude of the local velocity vector. Note the beginning of streamlines at the lateral cylinder surface, reflecting the distribution of osmotic water sources, and the steep velocity increase towards the open outlet. As flow is observed continuously all the way from the very tip of the channel, we will below introduce the term “wet-tip regime” for the underlying parameter choices. When repeating the simulation for different parameter values, then also a different flow regime is encountered in which flow ceases in a self-organized manner near the tip of the channel, see Fig. 1B’. Below, we will term this regime “dry-tip” as no fluid enters the channel at steady state for some portion downstream of the tip. The data from our numerical analysis of the three-dimensional problem will be discussed in a corresponding section below. We here aim to understand the transition between these flow patterns as well as their properties and parameter dependencies.

Towards analytical analysis, we first derive a one-dimensional model of coupled fluid and osmolyte transport by considering (i) flow in a narrow channel (blue arrows in Fig. 1A), (ii) osmotic driving and pressure feedback (upper red arrows in Fig. 1A) and (iii) the mass-balance of osmolytes (green and lower red arrows in Fig. 1A). A one-dimensional model here captures all important aspects since the cross-sectional Péclet number is of the order ${10}^{-4}\ll 1$ for relevant systems, see next Section and Methods. The derivation is given in a Methods section and yields coupled equations for the cross-section averaged velocity profile $\overline{w}(z,t)$ following Darcy’s law, the pressure profile $p(z,t)$ in the continuity equation and the osmolyte concentration profile $c(z,t)$ governed by mass-balance:

1 $$\overline{w}(z)=-\,\frac{{a}^{2}}{8\mu }\frac{\partial p(z)}{\partial z},$$

2 $$\frac{\partial \overline{w}(z,t)}{\partial z}=\frac{2\kappa }{a}[RT\,c(z,t)-p(z,t)]$$

and

3 $$\frac{\partial c(z,t)}{\partial t}=\frac{2}{a}g(z,t)+{D}_{zz}\frac{{\partial }^{2}c(z,t)}{\partial {z}^{2}}-\frac{\partial }{\partial z}(c(z,t)\overline{w}(z,t)).$$

Mixed boundary conditions are defined with no-flux at $z=0$ for solvent ($\overline{w}(z=0)=0$) and solute $({\frac{\partial c(z,t)}{\partial z}|}_{z=0}=0)$, and zero pressure at the outlet ($p(z=L)=0$). A summary of all model components and parameters is given in Table 1.

**Table 1:** Notation of model variables and parameters.

Symbol	Description
*t*	Time
${\bf{x}}=\{r,\varphi ,z\}$	Cylindrical coordinate system anchored at the symmetry axis of the channel
${\bf{u}}=\{u,v,w\}$	Fluid velocity vector in the cylindrical coordinate system
$\overline{w}(z)$	Cross-section average of axial fluid velocity
*p*(**x**)	Hydrodynamic fluid pressure
*p*_*osm*_(**x**)	Osmotic pressure induced by the solute
*j*(**x**)	Osmotic fluid influx density across channel membrane
*c*(**x**)	Osmolarity of the fluid
*D*	Solute diffusion coefficient
*g*(**x**)	Solute secretion rate per unit membrane area
*a*	Radius of the channel
*L*	Length of the channel
*κ*	Water permeability of channel membrane
*μ*	Dynamic viscosity of the fluid
*R*	Universal gas constant
*T*	Fluid temperature

A special case of this general model has recently been applied to bile flow in the mouse liver and was validated using live *intra-vital* microscopy data^25^. Therein, specific parameter values inferred from untreated mice together with measured solute secretion upon treatment allowed to correctly predict bile flow in treated mice. Complementing these studies, we here aim at understanding the model’s behavior, notably different flow regimes, for any parameter combination and also including heterogeneous osmolyte concentration profiles.

### Mapping of the general steady state problem to an Abel equation and analytical solution for limit cases

To gain analytical insight into parameter dependencies, we aim at a closed-form solution for the pressure profile and concomitant velocity and water influx profiles. In Eq. 3, a potential time dependency in $c(z,t)$ can only result from time-dependent solute secretion $g(z,t)$, which then renders all other variables of the fluid dynamics problem time-dependent. However, for temporally constant solute secretion *g*(*z*) and as a consequence of the saturating role of the negative feedback loops without time delays (see Fig. 1A), both the fluid flow and the concentration profile converge to a stable steady state, which is reproduced with high accuracy by our numerical simulations (see Fig. 1B,C). We are restricting our analytical analysis to this steady state. Additionally, one can account for slow time dependencies in parameters or solute secretion $g(z,t)$ by adiabatic approximation and substitution of slow time dependencies into the solutions derived here.

Moreover, there are two regimes of the axial Péclet number (Pé = $L\overline{w}$/*D*) in which either diffusion (Pé $\ll$ 1) or advection (Pé $\gg$ 1) is the dominant transport mechanism of osmolytes such that ongoing secretion with positive *g*(*z*) gets balanced. The range of different secretory systems with channel lengths between 10 *μ*m (lateral cleft in epithelia) and 1mm (intrahepatic bile canaliculi or pancreatic ducts), with flow velocities around the *μ*m/s range^25^ and with diffusion constants between 10 *μ*m^2^/s (large molecules like bile salts) and 10^3^ *μ*m^2^/s (K^+^ ions in water) results in Péclet numbers that span across both regimes and we therefore consider both regimes. The regime of dominating diffusion (Pé $\ll$ 1), leading to uniform $c=const$ and allowing to derive a general solution for any choice of *g*(*z*), will be analyzed in a subsequent section below while mixed cases of comparable contributions by diffusion and advection (Pé~1) can be studied numerically. Here, we first consider the more difficult regime of dominating advection (Pé $\gg$ 1) over diffusion and analyze Eq. 3 without the diffusion term and at steady state:

4 $$\frac{\partial c(z)}{\partial t}=0=\frac{2}{a}g(z)-\frac{\partial }{\partial z}(c(z)\overline{w}(z)).$$

Taking the definite integral with respect to the axial coordinate from 0 to *z* on both sides of Eq. 4, we can solve for solute concentration *c*(*z*) at steady state:

5 $$c(z)=\frac{G(z)}{\overline{w}(z)}=\frac{G(z)}{-\frac{{a}^{2}}{8\mu }\frac{dp(z)}{dz}},$$

where $G(z)=\frac{2}{a}\,{\int }_{0}^{z}\,g(z^{\prime} )dz^{\prime}$ represents the cumulative upstream solute secretion flux and Eq. 1 has been inserted to relate *c* and *p*. Substituting *c*(*z*) from Eq. 5 into Eq. 2, yields a single nonlinear ordinary differential equation for *p*(*z*):

6 $$\frac{{d}^{2}p(z)}{d{z}^{2}}=-\frac{16\kappa \mu }{{a}^{3}}[RT\frac{G(z)}{-\frac{{a}^{2}}{8\mu }\frac{dp(z)}{dz}}-p(z)].$$

To appreciate the fundamental scaling relations between the physical quantities, we partially non-dimensionalize Eq. 6 by introducing the relative coordinate $\tilde{z}$ and the non-dimensional parameter grouping *M*:

7 $$\begin{array}{rcl}\tilde{z} & = & \frac{z}{L}\in [0,1],\\ M & = & \frac{2\pi aL\kappa }{\frac{\pi {a}^{4}}{8\mu L}}=\frac{16\kappa \mu {L}^{2}}{{a}^{3}},\end{array}$$

that represents the ratio of osmotic conductivity for water (membrane surface area times membrane permeability) to hydrodynamic conductivity and is known as the Münch number^26^. Eq. 6 then reads:

8 $$\frac{{d}^{2}p}{d{\tilde{z}}^{2}}(\tilde{z})=M[\frac{8RT\mu L}{{a}^{2}}\frac{G(\tilde{z})}{\frac{dp}{d\tilde{z}}(\tilde{z})}+p(\tilde{z})].$$

We first consider two limit cases of small and large Münch number:

1. Letting $M\ll 1$ gives $\frac{{d}^{2}p}{d{\tilde{z}}^{2}}(\tilde{z})=const\ll 1$, then integration yields
  9 $$p(\tilde{z})={P}_{2}\frac{{\tilde{z}}^{2}}{2}+{P}_{1}\tilde{z}+{P}_{0},\,{P}_{0,1,2}=const.$$
  Determining ${P}_{1}=0$, ${P}_{2}=-\,2{P}_{0}$ from the boundary conditions irrespective of the osmolyte secretion profile, yields the pressure profile as an inverted parabola ~$(1-{\tilde{z}}^{2})$ and the velocity profile as linear. Hence unobstructed flow occurs from the closed tip onward for all parameter combinations corresponding to a small Münch number.
2. In the case when $M\gg 1$ and for physiological pressure profiles with bounded values and derivatives, the value of the bracket in Eq. 8 has to vanish. Reverting the substitution of *c*(*z*) from Eq. 5 for this bracket, we obtain

10 $$0=-\,RT\,c(\tilde{z})+p(\tilde{z}).$$

Hence, the right hand side in Eq. 2 becomes zero and the gradient of the velocity vanishes, keeping the velocity at its boundary value $w=0$. So, all parameter combinations corresponding to a large Münch number prevent normal outflow in agreement with the definition of *M*.

For arbitrary *M*, we can integrate Eq. 8 with respect to $\tilde{z}$ and obtain a special nonlinear ordinary differential equation of Abel type (Eq. 26), see Methods, for which particular solutions are known. However, Eq. 26 still poses difficulties for finding the general solution given an arbitrary solute secretion profile $g(\tilde{z})$. Hence, we consider particular profiles $g(\tilde{z})$ and then identify commonalities and differences between the solutions of relevant particular cases.

First, we choose the case that yields uniform concentration $c(\tilde{z})=\overline{c}$, which in the following section permits us to look for the pressure solution $p(\tilde{z})$ and corresponding solute secretion profile $g(\tilde{z})$ independently. Then we extend this approach to non-homogeneous osmolyte profiles $c(\tilde{z})$ in the subsequent section.

### Analytical solution for uniform osmolyte concentration

We let $RT\overline{c}={p}_{osm}=const$ in Eqs 2 and 8, and apply the non-dimensionalization from Eq. 7, to get

11 $$\frac{{\partial }^{2}p(\tilde{z})}{\partial {\tilde{z}}^{2}}-M\,p(\tilde{z})+M\,{p}_{osm}=0,$$

which has the general solution $p(\tilde{z})={\sum }_{i}\,{A}_{i}{e}^{{B}_{i}\tilde{z}}$, with ${A}_{i},{B}_{i}=const$. Fixing *A*_*i*_, *B*_*i*_ such that the mixed boundary conditions $({\frac{dp}{dz}|}_{\tilde{z}=0}=0,p(\tilde{z}=1)=0)$ are fulfilled, yields the solution:

12 $$p(\tilde{z})={p}_{max}\frac{\cosh \,\sqrt{M}-\,\cosh \,\sqrt{M}\tilde{z}}{\cosh \,\sqrt{M}-1}\mathrm{.}$$

This pressure profile is monotonously decreasing (solid curves in Fig. 1C,C’) with its maximum *p*_*max*_ located at the closed tip ($\tilde{z}=0$):

13 $${p}_{max}={p}_{osm}\frac{\cosh \,\sqrt{M}-1}{\cosh \,\sqrt{M}}\le {p}_{osm}.$$

Hence the maximum hydrostatic pressure, which could potentially damage cells, is intrinsically limited by the osmotic pressure. This intrinsic limitation may safeguard the surrounding cells, that establish the osmotic pressure by actively pumping osmolytes into the lumen, as higher hydrostatic pressures interfering with cell geometry and cell functions would reduce the cells’ pump efficiency and cause both pressures to relax. Another characteristic of the solution is the spatially averaged pressure as a function of Münch number *M*:

14 $$\langle p\rangle (M)={\int }_{0}^{1}\,p(\tilde{z})\,d\tilde{z}={p}_{{\max }}\frac{\sqrt{M}\,\cosh \,\sqrt{M}-\,\sinh \,\sqrt{M}}{\sqrt{M}(\cosh \,\sqrt{M}-1)}.$$

**Figure 2:**
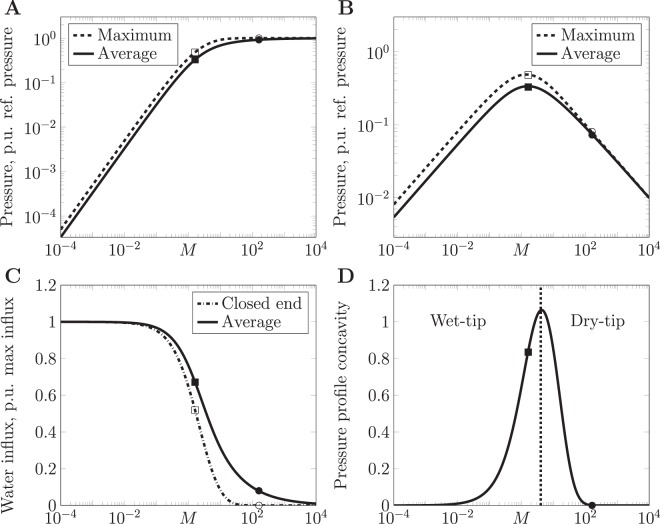
Parameter dependencies reveal two flow regimes. (**A**,**B**) Maximum and spatial average of pressure $p(z)={p}_{osm}(\kappa )\cdot \tilde{p}(M,z)$ from Eqs 13 to 15 and 30 are evaluated as function of the non-dimensional Münch number $M=\frac{16\,\kappa \mu {L}^{2}}{{a}^{3}}$ for fixed maximum osmolyte secretion *g*_0_, in (**A**) normalized by *p*_*osm*_$(\kappa )$ and in (**B**) as function of permeability *κ* plotted over $M(\kappa )$ and normalized to a *κ*-independent ${p}_{osm}^{\mathrm{(0)}}$ from Table 2. (**C**) Water influx *j* as function of *M* from Eqs 31 and 32 shows a transition between uniform influx and drying-up. (**D**) The modulus of the pressure profile concavity $|{\partial }^{2}p/\partial {\tilde{z}}^{2}|$ at the closed tip shows a marked maximum at the regime transition *M*_*cr*_ (see Eq. 18, marked with a dotted line). Throughout, *c* is uniform and squares (circles) denote solutions in Fig. 1C,C’, parameter values in Table 2.

Figure 2A,B show these dependencies of the fluid pressure on the non-dimensional parameter grouping *M*. Moreover, Taylor expansion of Eq. 12 reveals linear dependencies ${p}_{max}={p}_{osm}M/2+{\mathscr{O}}({M}^{2})$ and $\langle p\rangle ={p}_{osm}M/3+$ ${\mathscr{O}}({M}^{2})$ for $M\ll 1$ and a plateau ${p}_{max}=\langle p\rangle ={p}_{osm}$ at $M\gg 1$, both in agreement with the limit cases studied for the general case above. Notably, these qualitatively different limits, one with strong versus the other with vanishing fluid secretion, indicate the existence of two different flow regimes.

Calculating the osmolyte concentration from given maximum osmolyte secretion, see Methods, and inserting this relation Eq. 30 into Eq. 13 gives the emergent pressure range:

15 $${p}_{max}=\sqrt{\frac{RT{g}_{0}}{\kappa }}\frac{\cosh \,\sqrt{M}-1}{\cosh \,\sqrt{M}},$$

where the pressure drop between the system boundaries (*p*_*max*_) is a model result and depends on the solute secretion rate as $\sqrt{{g}_{0}}$ as well as all other parameters, whereas in previously studied fluid flow models a pressure drop between the system boundaries had to be pre-defined.

This model prediction reveals a non-trivial pressure dependence on the membrane permeability *κ*, exhibiting non-monotonic behavior, as *κ* affects the pressure explicitly and implicitly through *M*. Figure 2B shows this increasing pressure at low permeability values, when high water influx steepens the pressure gradient, versus decreasing pressure at high permeability values, when larger water inflow washes away the osmolyte and prevents its accumulation and concomitant pressure build-up.

As a consequence of conservation of fluid volume, we consistently observe that the cumulative inflow $L\cdot \langle j\rangle =\pi {a}^{2}\overline{w}(\tilde{z}=1)$ equals the terminal fluid outflow, linking Eq. 32 with Eq. 27, see Methods. In Fig. 2C we observe that the average water influx $\langle j\rangle$ vanishes for large Münch number and hence the overall outflow of the channel stalls ($\overline{w}(\tilde{z}=1)\to 0$). The transition between the unobstructed flow regime at low Münch number and this stalled-flow regime is investigated in a subsequent section.

### General solution for dominating osmolyte diffusion

Complementary to the above case of dominating advective transport over osmolyte diffusion, we next analyze the case (Pé $\ll$ 1) of osmolyte diffusion dominating over advective transport and heterogeneities in osmolyte secretion. Eq. 3 then describes the relaxation towards a stable uniform concentration state with $c(z,t)=\overline{c}$. Hence all derivations and solutions from above, i.e. Eqs 11 to 15, apply here as well and, moreover, they apply for arbitrary forms of *g*(*z*) as long as diffusion dominates. The uniform concentration $c(z,t)=\overline{c}$ is then established as balance between cumulative secretion and terminal outflow. Correspondingly, integrating Eq. 3 over the channel volume and inserting $\overline{w}(\tilde{z}=1)$ from Eq. 27 yields:

16 $$\overline{c}=\sqrt{\frac{\sqrt{M}}{RT\,\kappa \,\tanh (\sqrt{M})}\,{\int }_{0}^{1}\,g(\tilde{z})d\tilde{z}}.$$

### Simulation results

To test the validity of our one-dimensional projection, the analytical predictions were compared to results from the three-dimensional numerical simulations (see Fig. 1B,B’ and Methods) by superimposing the fluid pressure, cross-section-averaged velocity, solute secretion rate and water influx profiles along the channel (see Fig. 1C,C’). Axis scales in Fig. 1C,C’ are set by the maximum values of the analytical prediction. Comparison shows that the analytical prediction matches the full-scale simulation well. However, near the open outlet where the osmotic water sources at the boundary are strongest and hence the three-dimensional flow profile deviates from a Poiseuille profile, the analytical prediction slightly overestimates the cross-section-averaged velocity of the three-dimensional solution, see second graph in Fig. 1C. We conclude that this combined numerical and analytical approach is consistent and extend it to more complex cases in the following sections.

### Retrograde flow for heterogeneous osmolyte concentration profiles

**Figure 3:**
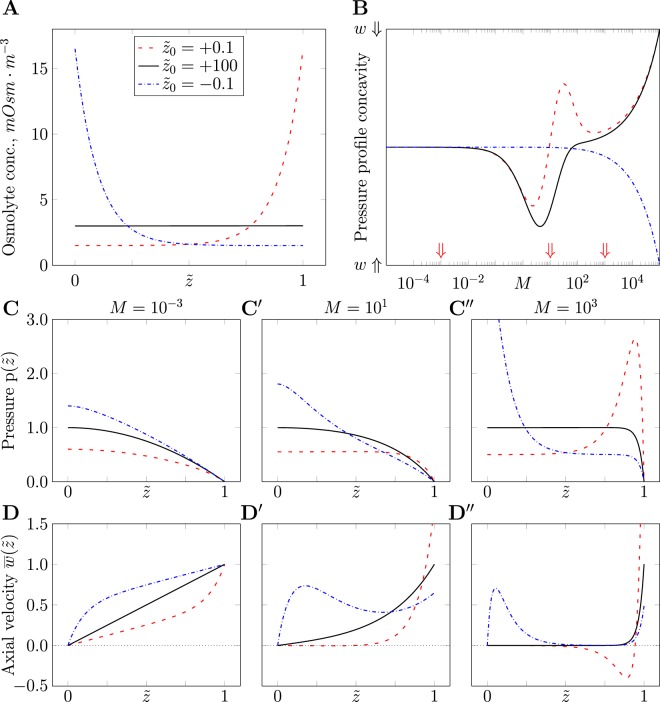
Solutions for heterogeneous osmolyte concentration profiles. (**A**) Considered qualitatively different concentration profiles (Eq. 17) with identical spatial averages $\langle c(\tilde{z})\rangle =\overline{c}$, implicitly fixing *c*_2_ given ${\tilde{z}}_{0}$ as in the legend and ${c}_{1}=\overline{c}/2$, parameter values as in Table 2. (**B**) Variation of pressure concavity ${\partial }^{2}p/\partial {\tilde{z}}^{2}$ from Eq. 34 at the closed tip with respect to Münch number *M*. The sign of the pressure concavity (+ in upper, −in lower half) determines whether the velocity is positive and grows towards the outlet (in lower half, *w*$\Uparrow$ symbol on the y-axis) or is negative (retrograde flow in upper half, *w*$\Downarrow$ symbol). Three qualitatively different cases considered in panels (**C**,**D**) are marked by red arrows. (**C**,**D**) Solutions $p(\tilde{z})$ ((**C**) Eq. 34) and $\overline{w}(\tilde{z})$ ((**D**) Eq. 35) for $M={10}^{-3}$ (left column), $M={10}^{1}$ (middle) and $M={10}^{3}$ (right) share the same axes in each row. In each panel, profiles are normalized with respect to the maximal value of that panel’s profile for $c(\tilde{z})=\overline{c}$. Throughout, line styles denote ${\tilde{z}}_{0}$ as in (**A**).

In hyper-osmotic secretory processes, an osmolyte concentration gradient along the channel is observed that combines water secretion with re-absorption^21^. We therefore consider the case of an exponentially distributed osmolyte concentration profile at steady state:

17 $$c(\tilde{z})={c}_{1}+{c}_{2}\,\exp \,(\frac{\tilde{z}}{{\tilde{z}}_{0}}),$$

where *c*_1_, *z*_2_ and ${\tilde{z}}_{0}$ are parameters (see Fig. 3A).

This case is solved in Eqs 34 to 36, see Methods. To study this solution, we consider the pressure profile concavity at the closed end (see Fig. 3B) and determine the Münch number ranges with characteristic behaviors of the system. We study several qualitatively different spatially-inhomogeneous osmolyte concentration profiles, but all with the same spatial average to ease comparison, see Fig. 3A.

Figure 3B shows that the behavior of the solution changes with the Münch number. We choose specific *M* values that show different trends of pressure profile concavity and, thereby, cause qualitatively different profiles for pressure, axial velocity and osmotic water influx, see Fig. 3C,D”. All shown spatial profiles are normalized by the respective maximal values for a uniform osmolyte concentration at the same Münch number, see previous section. This comparison shows that by varying fluid properties and channel geometry, one may achieve qualitatively different behaviors of the system, ranging from fluid secretion to its re-absorption.

In particular, a large decay length (black curves in Fig. 3A,C,D) yields spatial profiles similar to those observed in the uniform concentration case (see Fig. 1C). A more pronounced spatial inhomogeneity in the osmolyte profile causes a significant change in the flow profile compared to the uniform case. A rising osmolyte concentration profile with a short length scale generates retrograde flows to the closed end and a steep rise in the velocity profile at the outlet, peaking much higher than the uniform case (red curves in Fig. 3A,C,D). This fluid velocity profile results from the steep rise in the osmotic water influx close to the outlet. Monotonously decreasing osmolyte concentration profiles never cause retrograde flows but may create a water re-absorption region in some part of the channel (blue curves in Fig. 3A,C,D).

### Wet-tip versus dry-tip regimes

Two different flow regimes exist in general, one at low versus another at high Münch number, as observed in Eq. 9 versus Eq. 10. As shown in Fig. 2A, the maximum pressure reaches a plateau at the osmotic pressure *p*_*osm*_ for large *M*. At spatial locations with $p(\tilde{z})={p}_{osm}$, osmotic driving is then counter-balanced and thereby abrogated, drying up the water influx. Also the average pressure approaches the same plateau, hence the spatial region with $p(\tilde{z})={p}_{osm}$ extends from the closed tip downward with further increasing *M*. This dries up water inflow at the closed tip and some region downstream from it (see Fig. 2C) and creates a steep pressure gradient near the open outlet (see Fig. 1B’,C’). This steep pressure gradient is departing from the pressure plateau at a position of highest concavity of the pressure profile. For decreasing *M*, the pressure plateau vanishes when this position of highest concavity has reached the closed tip $\tilde{z}=0$. This prompts us to determine the critical value *M*_*cr*_ of the flow regime transition from the maximum curvature of the pressure profile (Eq. 12) at $\tilde{z}=0$, as shown in Fig. 2D. Setting the derivative of this dependence with respect to *M* to zero yields:

18 $${M}_{cr}=4\,{(\coth \sqrt{{M}_{cr}})}^{2}\approx 4.$$

Importantly, this result is independent of other system parameters including the osmotic pressure *p*_*osm*_. The particular value ${M}_{cr}\approx 4$ is a result of the considered feedback structure (see Fig. 1A) and governing equations (Eqs 2 and 3). From the definition of *M* (Eq. 7), we predict that the osmotic conductivity of the channel surface must remain smaller than 4 times the hydrodynamic conductivity of the channel to be able to completely drain the channel.

We now introduce the term “wet-tip regime” for an unobstructed steady state outflow from the very tip of the channel. This wet-tip regime occurs for $M < {M}_{cr}$. Conversely, the term “dry-tip regime” will be used for stalled flow at the very tip of the channel and some part of the channel downstream of the tip. The term “dry” serves to indicate the lack of continuous water influx despite ongoing osmolyte secretion. This dry-tip regime occurs for $M > {M}_{cr}$. The surface $M(\kappa ,\mu ,a,L)={M}_{cr}$ separates the wet-tip from the dry-tip regime in the 4-dimensional parameter space. Using the definition of *M* in Eq. 7, it is then straight forward to decide, for a given set of geometrical and material parameters, whether a channel can be drained all the way from its tip on.

**Figure 4:**
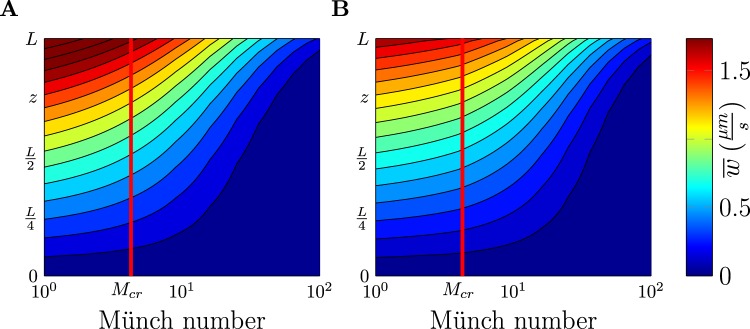
Wet-to-dry tip transition is similar for different osmolyte secretion cases. (**A**,**B**) Velocity profiles $\overline{w}(z)$ from 3D numerical simulations, color-coded and *z* on vertical axis, as function of Münch number *M* for $c=const$ (**A**) and $g=const$ (**B**). Also, data indistinguishable from (**A**) is obtained from the one-dimensional analytical solution in Eq. 27 and applies to any osmolyte secretion profile *g*(*z*) at low Péclet number. For all cases, *M*_*cr*_ (vertical red line) separates the wet-tip regime with unobstructed flow at $M < {M}_{cr}$ from the dry-tip regime with stalled flow in part of the channel at $M > {M}_{cr}$. In the limits $M\ll 1$ and $M\gg 1$, the solution is known analytically for any case, see Eqs 9 and 10.

The analytical results obtained above were derived for the case of uniform osmolyte concentration, corresponding to spatially increasing osmolyte secretion rate $g(\tilde{z})$. An analogous result for a stagnant zone was derived by Rademaker *et al*.^20^. To verify the broader applicability of the criterion $M={M}_{cr}$ for the regime boundary, we numerically solved the complementary case of uniform osmolyte secretion rate $g(\tilde{z})=const$ as presented in Fig. 4 and found a qualitatively similar transition. Details of the numerical analysis are given in the Methods section. These analytical and numerical results together with the limiting solutions (Eqs 9 and 10) establish the wet-tip versus dry-tip transition as a general phenomenon.

### Applications of the theory

As a quantitative test of our theory, it has recently been applied in an *intra vital* microscopy study of fluorescent tracer transport with bile flow in murine liver^25^. Parameter estimation for normal control mice and model predictions had been performed analytically and numerically using the simulation software Morpheus^27^. Comparison of model predictions for treatment with the commonly used analgesic acetaminophen (APAP) to experimental data had confirmed the model and identified an additional contractile driving mechanism especially in a zone near the closed tip. Potentially, this additional mechanism safeguards the mouse liver against the dry-tip regime as the Münch number calculated from their data (parameters ${p}_{2}=16\,\kappa \mu ={6.9710}^{-6}\,\mu m$, $L=344\,\mu m$, $a=\langle {a}_{a}(x)\rangle {r}_{corr}=0.41\,\mu m$ reported in^25^) yields $M={p}_{2}{L}^{2}/{a}^{3}\,\approx \,12$ and therewith slightly exceeds *M*_*cr*_ calculated here. This inferred Münch number is consistent with the strong curvature of the experimentally measured velocity profile.

On the other hand, typically wider channel radii in other systems will, with the third power of the radius, lead to smaller Münch numbers and we evaluate Eq. 7 for two such systems. For the classical problem of phloem flow in a plant leaf, we consider $a=10\,\mu m$, $L=10\,mm$, $\mu =1.5\,mPa\cdot s$, $\kappa =2\cdot {10}^{-5}\,\mu m/(Pa\cdot s)$ following Table 3 in^16^. The resulting Münch number $M=0.048$ is below *M*_*cr*_, confirming free flow^16^.

For the kidney, models of osmotic fluid transport have addressed the question of urine concentration in the nephron due to water uptake from the nephron into the interstitium^28–30^. While the geometry of the interstitium is different from the tube considered here, a similar flow problem and dry-tip regime may in principle arise inside the interstitium. To estimate the Münch number for such a geometry around a nephron in rat, we consider $a\,\sim \,10\,\mu m$, $L\,\sim \,1\,mm$, $\mu =0.7\,mPa\cdot s$ (viscosity of water at 37 °C) and membrane permeability *κ* for water at different positions in the range $(0.6\ldots 516.8)\cdot {10}^{-5}\,\mu m/(Pa\cdot s)$ derived from experimental data (last column of Table 2 in^31^ and assuming a typical osmotic pressure from^16^). The resulting range of Münch numbers spans $7\cdot {10}^{-5}$ to 0.06 below *M*_*cr*_, predicting free flow in the kidney.

### Model implications for organ-size

Continuous drainage of the whole channel and thereby avoiding hyper-concentration and aggregation of solutes is of high importance for maintaining proper function of any secretory organ. Hence, it is further useful to derive the largest channel length that can still be drained completely by osmotic driving, so we invert Eq. 7 and obtain

19 $${L}_{max}=\sqrt{\frac{{M}_{cr}}{16}\frac{{a}^{3}}{\kappa \mu }}.$$

Should the channel length increase beyond *L*_*max*_ then the dry-tip regime is reached for the channel region $z\in [0;L-{L}_{max}]$, while the part of length *L*_*max*_ near the open outlet sustains unobstructed outflow. Secretory organs may rely on this transition to control their size during growth in development and regeneration. Analogously, the maximum observed size for plant needles of around 5 *cm* has been explained^20^.

## Discussion

Here we studied the biophysical effects of osmotic driving during fluid secretion using a mathematical modeling approach. Opposite to many fluid dynamics problems with given pressure gap across the system boundaries, here the pressure gap is an emergent property of the feedback loops in the system. Our numerical and analytical results have revealed the relation between tissue geometry and rheological properties of the secreted fluid and the function of secretory organs. The results identify two fundamental flow regimes: a free outflow from all the length of the channel, termed wet-tip regime, and a combination of free outflow with ceased flow within some portion of the channel near the closed end, the dry-tip regime.

The transition was found to depend on the value of a non-dimensional parameter grouping, the Münch number $M=16\,\kappa \mu {L}^{2}/{a}^{3}$. In particular for $M\gg 1$, the total outflow $\pi {a}^{2}\overline{w}(L)\,\sim \,({a}^{4}\sqrt{M}/(\mu L))\,\ast \,\tanh \,\sqrt{M}$ from the channel outlet yields $\sqrt{{a}^{5}\kappa /\mu }$ independent of *L*. This refutes intuition stemming from approximated uniform osmotic inflow that would cumulatively grow with *L* as ~*κaL*. The above counterintuitive parameter dependency originates from the competition of osmotic pressure against hydrostatic pressure (see pressure difference in Fig. 1A) for water flux through transmembrane water channels. This competition limits the functional part of the channel to the downstream portion of length *L*_*max*_ while the upstream portion suffers the dry-tip condition. For comparison, an oversimplified model neglecting the pressure feedback (no pressure difference in Fig. 1A) would yield the predictions of the wet-tip regime for any parameter combination (Münch number) and miss the dry-tip regime.

By considering heterogeneous osmolyte concentration profiles, we have explored how osmosis sustains steady and spatially stratified patterns of water secretion and re-absorption. Our results to lowest order also extend to systems with weakly heterogeneous parameter profiles, *a*(*z*), *κ*(*z*) or *μ*(*z*), where these functions can be substituted into our pressure and velocity solutions and their spatial average enters the Münch number. From a methodological perspective, the deeper understanding of physiological behavior was obtained by the close integration of numerical and analytical methods as opposed to just applying numerical simulations alone. This integrated approach has been fruitfully applied before to questions of signaling, spatio-temporal patterning and function in liver cells^32^, pancreas^33^ and tissue regeneration^34^.

We next explore implications of our theory for bile flow in the liver. Pathologies of bile flow range from solute aggregation into gallstones via stalled flow in cholestasis to bile infarct at too high bile fluid pressure^24^. Although recent theoretical work has considered bile flow in larger non-secreting ducts downstream of the gallbladder^35–37^, feedback-controlled bile formation as studied here was not part of that work. Using our Eq. 19, we can now predict a critical canalicular path length beyond which the dry-tip regime sets in. Then, the flow in part of the channel $z\in [0,L-{L}_{max}]$ is stalled, which is reminiscent of cholestasis^24^. Our model suggests that the dry-tip regime may arise either due to reduction in the width of the channel (e.g. as in biliary constriction) thereby reducing ${L}_{max}\,\sim \,{a}^{\mathrm{3/2}}$ or by increasing the viscosity of bile (e.g. as in Byler disease) thereby reducing ${L}_{max}\,\sim \,{\mu }^{-\mathrm{1/2}}$. A gradual parameter shift over the course of disease progression corresponds to a continuous increase of *M* and Fig. 4 shows the resulting flow re-distribution.

As a subject of future work on more realistic channel geometries, it will be interesting to consider bile canaliculi in the vertebrate liver as an interconnected network including closed loops of branches. Such closed loops will result in many local pressure maxima and corresponding flow stagnation points, thereby reducing the potential output of the system while gaining robustness against perturbation of individual branches. Potentially, the stable fluid flow solutions with osmotic driving in looped network architectures are no longer restricted to stationary states as for the unbranched channel considered herein. These extensions may also connect to results for flow routing in capillary networks^38,39^. Whether channel geometry and fluid properties are actively adjusted to the capacity of osmotic driving in order to sustain an outflow from the channel’s closed tip remains an interesting open question which can now be considered in the light of the wet-to-dry tip transition. Finally, it will be interesting to survey a broader set of secretory systems to compare their physiological range of Münch numbers and relate these to the threshold at ${M}_{cr}\approx 4$.

## Conclusions

We developed a mathematical model of the coupled osmolyte transport and fluid secretion problem and considered its steady-state dynamics. The obtained solutions for both homogeneous and inhomogeneous osmolyte distributions reproduce physiologically relevant flows. Our analytic results revealed a wet-to-dry tip transition between physiological outflow and pathological stalled-flow conditions based on a ratio between four parameters of the system, known as the Münch number $M=16\,\kappa \mu {L}^{2}/{a}^{3}$. The derived criterion $M > {M}_{cr}\approx 4$ gives a theoretical prediction for the onset of pathological conditions in vertebrate glands, like cholestasis in the liver, and may set a physical limit on organ size during growth and regeneration. Numerical simulations of the full-scale model confirmed these analytical predictions.

## Methods

### Model derivation

Fluid flow in long and narrow cylindrical channels allows for approximations to the Navier-Stokes equations. Here, ${\bf{u}}({\bf{x}})=(u,v,w)$ is the fluid velocity in cylinder coordinates, *p*(*z*) is the fluid pressure and *μ* is the dynamic viscosity. We consider the steady laminar flow that preserves the axial symmetry of the channel geometry since the Reynolds numbers for the considered biological systems are low, e.g. for bile flow it was estimated as Re~10^−6^ which lies 9 orders of magnitude below the threshold to turbulent flow^25^. Also, for $a\ll L$ we can neglect the radial components of the fluid velocity, thereby reducing the problem to one spatial dimension. As it then suffices to analyze the cross-section average $\overline{w}$ of the axial velocity component, we set it proportional to the negative local pressure gradient, following Darcy’s law

20 $$\overline{w}(z)=-\,\frac{{a}^{2}}{8\mu }\frac{\partial p(z)}{\partial z}.$$

Here, the pre-factor is obtained by integrating the axial fluid velocity component of Poiseuille flow in the cylindrical channel with respect to the radial coordinate $(w(r,z)=-\,\frac{{a}^{2}-{r}^{2}}{4\mu }\frac{\partial p(z)}{\partial z})$, which may differ for other channel geometries. The solutions of this one-dimensional approximation agree with the results of three-dimensional computational fluid dynamics simulations, see Fig. 1C,C’ and results below.

Second, the considered dilute solution of osmolytes allows to expand the osmotic pressure *p*_*osm*_(*c*) in powers of solute concentration, known as the virial expansion^40^. As a first-order approximation, one arrives at van’t Hoff’s law ${p}_{osm}=RTc$^41^, where *R* is the universal gas constant, *T* is the temperature in degree Kelvin and *c*(*z*, *t*) is the solute concentration with reflection coefficient 1. The fluid influx *j* is proportional to the difference between the osmotic driving Δ*p*_*osm*_ and the hydrodynamic pressure Δ*p* across the channel surface (Δ $\stackrel{\wedge}{=}$ intraluminal value minus value in surrounding tissue)^20,40,42,43^:

21 $$j=\kappa \,({\rm{\Delta }}{p}_{osm}-{\rm{\Delta }}p),$$

where *κ* is the membrane permeability (among others, proportional to the aquaporin density in the surrounding cell membrane and the leakiness of cell-cell junctions). Then, the continuity equation for any infinitesimal control volume yields

22 $$\frac{\partial \overline{w}(z,t)}{\partial z}=\frac{2}{a}j(z,t)=\frac{2\kappa }{a}\,[RT\,c(z,t)-p(z,t)],$$

where 2/*a* is the surface to volume ratio. The difference between osmotic and hydrodynamic pressures within the surrounding tissue is considered negligible against the difference of the intraluminal pressures.

Third, to account for the feedback of fluid dynamics onto osmotic driving, the mass-balance of osmolyte concentration *c*(*z*, *t*) is modeled as

23 $$\frac{\partial c(z,t)}{\partial t}=\frac{2}{a}g(z,t)+{D}_{zz}\frac{{\partial }^{2}c(z,t)}{\partial {z}^{2}}-\frac{\partial }{\partial z}(c(z,t)\overline{w}(z,t)),$$

where *g*(*z*) is the osmolyte secretion flux per surface area, *D*_*zz*_ is the osmolyte diffusion constant and the last term describes the advective wash out of osmolytes by the fluid flow. The concentration profile can be considered uniform in the lateral directions with extension $a\ll L$ since the lateral Péclet number scales with *a*^2^/*L*^2^ (~10^−6^ for a typical case, see Table 2) in relation to the longitudinal Péclet number and yields Pé_*lat*_ $\ll$ 1, see Results. Note, the position dependence of the velocity $\overline{w}(z,t)$ is essential in Eq. 3 as opposed to blood flow models. Mixed boundary conditions are defined with no-flux at $z=0$ for solvent ($\overline{w}(z=0)=0$) and solute $({\frac{\partial c(z,t)}{\partial z}|}_{z=0}=0)$, and zero pressure at the outlet ($p(z=L)=0$).

**Table 2:** Model parameter values (top rows) used in the simulations and predicted characteristic solution properties (two bottom rows, related to Fig. 1B,B’,C,C’).

Symbol	Value	Unit	Refs
*a*	$1.4\cdot {10}^{-6}$	m	^7^
*L*	10^−3^	m	^7^
*κ*	$30.2\cdot {10}^{-11}$ ^( *a*)^	${\rm{m}}\cdot {{\rm{s}}}^{-1}\cdot {{\rm{Pa}}}^{-1}$	^45^
*R*	8.3144621(75)	${\rm{J}}\cdot {{\rm{K}}}^{-1}\cdot {{\rm{mol}}}^{-1}$	
*T*	310	K	
*μ*	$0.92\cdot {10}^{-3}$ ^( *a*)^	${\rm{Pa}}\cdot {\rm{s}}$	^36^
$\overline{c}$	3.0^(*a*)^	${\rm{mOsm}}\cdot {{\rm{m}}}^{-3}$	
*D*	10^−6^	${{\rm{m}}}^{2}\cdot {{\rm{s}}}^{-1}$	
*M*_0_	1.6	dim-less	
${p}_{osm}^{(0)}$	7.7	Pa	

^(*a*)^Please see Methods for rationale behind choices of specific parameter values.

### Derivation of Abel equation for arbitrary Münch number

Combining Eq. 2 with Eq. 4 and substituting *G*(*z*), $\tilde{z}$, *M* from Eq. 7, we arrive at a model for the unknown pressure profile in Eq. 8, see Results. We can then integrate Eq. 8 with respect to $\tilde{z}$ and obtain the special nonlinear ordinary differential equation of Abel type:

24 $$\frac{1}{M}{(\frac{dp}{d\tilde{z}})}^{2}={\alpha }^{2}{\rm{\Gamma }}(\tilde{z})+{p}^{2}.$$

Where the pressure amplitude scales with the coefficient $\alpha =\sqrt{\frac{16\,\mu RTL}{{a}^{2}}}$ and the square root of ${\rm{\Gamma }}(\tilde{z})=$ $\int \,G(L\tilde{z})d\tilde{z}+{{\rm{\Gamma }}}_{0}$.

For mapping Eq. 24 to the canonical form of the Abel equation, we substitute $\xi (\tilde{z})=\alpha \sqrt{{\rm{\Gamma }}(\tilde{z})}$ and $p(\tilde{z})=\xi (\tilde{z})\,\sinh \,\psi (\tilde{z})$ and get

25 $$\frac{d\xi }{d\tilde{z}}\,\tanh \,\psi +\xi \frac{d\psi }{d\tilde{z}}\mp \sqrt{M}\,\xi =0.$$

The negative sign in front of the last term represents a rising pressure profile and, therefore, an inflow at the open outlet. However, we restrict our analysis to the physiologically relevant condition of fluid secretion with a pressure profile decreasing towards the open outlet, hence, we choose to consider only the case with the positive sign. This equation can be transformed into an Abel equation of the first kind^44^ with non-constant coefficients by letting $\theta (\tilde{z})=\,\tanh \,\psi (\tilde{z})$:

26 $$\xi \frac{d\theta }{d\tilde{z}}-\frac{d\xi }{d\tilde{z}}{\theta }^{3}+\sqrt{M}\,\xi {\theta }^{2}+\frac{d\xi }{d\tilde{z}}\theta -\sqrt{M}\,\xi =0,$$

for which particular solutions are known.

### Solution for influx profiles of osmolytes and water

We here derive the solution for the influx profile of osmolytes $g(\tilde{z})$ that yields the special case of uniform osmolyte concentration even for negligible osmolyte diffusion. We also summarize the corresponding solutions for the flow velocity profile $\overline{w}(\tilde{z})$ and the water influx profile $j(\tilde{z})$ that were derived previously^25^.

For the velocity profile, inserting Eq. 12 into Eq. 1 yields

27 $$\overline{w}(\tilde{z})=\frac{{a}^{2}}{8\mu }\frac{{p}_{osm}\sqrt{M}}{L}\frac{\sinh \,\sqrt{M}\tilde{z}}{\cosh \,\sqrt{M}}.$$

Typical velocity profiles are shown in Fig. 1C,C’ and reproduce an experimentally validated solution^25^.

Inserting this velocity profile into Eq. 4, we obtain the secretion rate profile that underlies uniform osmolyte concentration:

28 $$\frac{\overline{c}}{L}\frac{\partial \overline{w}(z)}{\partial \tilde{z}}=\frac{2}{a}g(\tilde{z}),$$

29 $$g(\tilde{z})={g}_{0}\frac{\cosh \,\sqrt{M}\tilde{z}}{\cosh \,\sqrt{M}}.$$

This function in monotonously increasing from $\frac{{g}_{0}}{\cosh \,\sqrt{M}}$ at the closed tip to its maximum *g*_0_ at the outlet (see Fig. 1C,C’). Note, for $M\ll 1$ the spatial variation becomes negligibly small and $g\approx const$. The solution (Eqs 12 and 29) is also fulfilling the Abel equation (Eq. 24) with ${{\rm{\Gamma }}}_{0}=-\,({g}_{0}{L}^{2}/(M{a}^{2}))\,({\cosh }^{2}\,(\sqrt{M})+1){/\cosh }^{2}\,(\sqrt{M})$.

Following from Eq. 28, the parameter *g*_0_ determines the value of the uniform solute concentration for any *M*:

30 $$\overline{c}=\sqrt{\frac{{g}_{0}}{\kappa RT}}.$$

An interesting observation is that $\overline{c}$ does not grow linearly with the secretion rate *g*_0_ but only with its square root due to the diluting negative feedback loop: osmolyte secretion *g*(**x**, *t*) affects the respective osmolyte concentration *c*(**x**, *t*), which in turn would change the pressure profile *p*(**x**, *t*) and, hence, the fluid velocity $\overline{w}({\bf{x}},t)$ and the latter again affects the osmolyte concentration *c*(**x**, *t*) and this closes the negative feed-back loop (see Fig. 1A).

Lastly, the heterogeneous and osmotically driven water influx density *j* (one-dimensional density per unit length) is obtained from Eq. 2 and reproduces the solution from^25^:

31 $$j(\tilde{z})=2\pi a\cdot \kappa \cdot {p}_{osm}\frac{\cosh \,\sqrt{M}\tilde{z}}{\cosh \,\sqrt{M}}.$$

For typical profiles, see Fig. 1C,C’). The spatially averaged fluid influx $\langle j\rangle$ is (see Fig. 2C):

32 $$\langle j\rangle \,(M)=2\pi a\cdot \kappa \cdot {p}_{osm}\frac{\tanh \,\sqrt{M}}{\sqrt{M}}.$$

### Analytical solution for special heterogeneous osmolyte concentration profiles

We consider the case of an exponentially distributed osmolyte concentration profile at steady state:

33 $$c(\tilde{z})={c}_{1}+{c}_{2}\,\exp \,(\frac{\tilde{z}}{{\tilde{z}}_{0}}),$$

where *c*_1_, *c*_2_ and ${\tilde{z}}_{0}$ are parameters (see Fig. 3A). Plugging Eq. 33 into Eq. 2 yields the pressure profile:

34 $$\begin{array}{rcl}p(\tilde{z}) & = & \frac{RT}{M{\tilde{z}}_{0}^{2}-1}\{\frac{\cosh (\sqrt{M}\mathop{z}\limits^{ \sim })}{\cosh (\sqrt{M})}\cdot [{c}_{2}\sqrt{M}{\tilde{z}}_{0}\,\sinh \,(\sqrt{M})\\  &  & -\,M{\tilde{z}}_{0}^{2}\,({c}_{2}{e}^{\frac{1}{{\tilde{z}}_{0}}}+{c}_{1})+{c}_{1}]\\  &  & -\,{c}_{2}\sqrt{M}{\tilde{z}}_{0}\,\sinh \,(\sqrt{M}\tilde{z})+M{\tilde{z}}_{0}^{2}\,({c}_{2}{e}^{\frac{\tilde{z}}{{\tilde{z}}_{0}}}+{c}_{1})-{c}_{1}\}.\end{array}$$

This profile does not possess a universally fixed position of its maximum (as opposed to the closed tip position in the case of uniform osmolyte concentration above) but this position varies as a function of the three parameters governing the osmolyte concentration profile. Such a non-monotonous solution may also describe a retrograde back-flow into the channel. To obtain the corresponding velocity profile, we substitute Eq. 34 into Eq. 1:

35 $$\begin{array}{rcl}\overline{w}(\tilde{z}) & = & \frac{{a}^{2}}{8\mu L}\frac{RT}{M{{\tilde{z}}_{0}}^{2}-1}\{\frac{\sinh \,(\sqrt{M}\tilde{z})}{\cosh \,(\sqrt{M})}\\  &  & \cdot \,[\sqrt{M}\,(M{\tilde{z}}_{0}^{2}({c}_{2}{e}^{\frac{1}{{\tilde{z}}_{0}}}+{c}_{1})-{c}_{1})-\,{c}_{2}M{\tilde{z}}_{0}\,\sinh \,(\sqrt{M})]\\  &  & +\,{c}_{2}M{\tilde{z}}_{0}\,\cosh \,(\sqrt{M}\tilde{z})-{c}_{2}M{\tilde{z}}_{0}{e}^{\frac{\tilde{z}}{{\tilde{z}}_{0}}}\}.\end{array}$$

Further, using Eq. 21, we can obtain the osmotic fluid influx profile along the channel:

36 $$\begin{array}{rcl}j(\tilde{z}) & = & \frac{2\pi a\kappa RT}{M{\tilde{z}}_{0}^{2}-1}\{\frac{\cosh \,(\sqrt{M}\tilde{z})}{\cosh \,(\sqrt{M})}\cdot [M{\tilde{z}}_{0}^{2}({c}_{2}{e}^{\frac{1}{{\tilde{z}}_{0}}}+{c}_{1})-\,{c}_{1}-{c}_{2}\sqrt{M}{\tilde{z}}_{0}\,\sinh \,(\sqrt{M})]\\  &  & +\,{c}_{2}\sqrt{M}{\tilde{z}}_{0}\,\sinh \,(\sqrt{M}\tilde{z})-{c}_{2}{e}^{\frac{\tilde{z}}{{\tilde{z}}_{0}}}\}.\end{array}$$

### Numerical solution of the three-dimensional full-scale problem

To study the system for arbitrary osmolyte secretion profiles *g*(*z*) and to test the applicability of the one-dimensional approximation for long and narrow channels with osmotic driving, numerical simulations of the coupled three-dimensional transport equations for water and solute were performed using the COMSOL software package (COMSOL Multiphysics^®^ v. 5.2, www.comsol.com, COMSOL AB, Stockholm, Sweden), see Fig. 1B,B’. The computational domain was defined as a cylinder with the following boundary conditions: no-flux at the closed tip; open-boundary at the outlet; solute secretion and solute-dependent water mass-inflow at the remaining boundaries.

For the case of uniform osmolyte secretion rate (see Fig. 4B), the osmolyte influx *g* is defined as fixed uniform source in the simulation. For the uniform concentration case (see Figs 1C,C’ and 4A), solute secretion was defined via a flexible source at the boundary that maintains a constant and homogeneous osmolyte concentration profile. The osmotic water influx density at the lateral boundaries is given by Eq. 21 and is used as input for the fluid dynamics solver. To avoid numeric convergence problems, $j\ge 0$ was set explicitly, corresponding to fluid secretion and outflow from the channel. The Stokes equations were coupled to the diffusion-advection equation for osmolyte transport and solved iteratively at steady-state on the whole computational domain using a direct solver. We have verified the numerical accuracy of the simulations by showing convergence of a solution metric for increasingly refined meshes (data not shown).

### Rationale for the choice of parameter values

Parameter values are given in Table 2 with their respective references when appropriate. Regarding the membrane water permeability per unit osmotic pressure, we estimated the order of magnitude as follows. A flux density $j=151\cdot {10}^{-6}\,{\rm{m}}\cdot {{\rm{s}}}^{-1}$ was measured^45^ for a fixed osmolyte gradient in shrinking apical vesicles obtained from primary hepatocytes of male Fisher rats. The permeability *κ* is obtained by dividing this flux density by the pressure difference. In this experiment, sucrose has been used as artificial osmolyte and we approximate the resulting osmotic pressure difference using the osmotic potential of a related and well-studied sugar (see Appendix A.3 in^16^), dextran, giving $\kappa =30.2\cdot {10}^{-11}\,{\rm{m}}\cdot {{\rm{s}}}^{-1}\cdot {{\rm{Pa}}}^{-1}$.

Regarding fluid viscosity, we considered bile as an example and assume that it is slightly more viscous than water, adopting the experimentally measured value for human hepatic bile^36^. Also note that under disease conditions, the viscosity of gallbladder bile^36^ may increase to a group median of $3\,{\rm{mPa}}\cdot {\rm{s}}$^46^. However, only gallbladder bile is directly accessible for collection and Luo *et al*. suggest that bile viscosity should be lower inside bile canaliculi than in the gallbladder^36^.

Regarding the osmotic driving, highly concentrated bile collected from the gallbladder yields $3\,{\rm{Osm}}\cdot {{\rm{m}}}^{-3}$^46^. Considering the much more dilute bile at the upstream level of bile canaliculi, for which no direct measurements exist, we here assume a three orders of magnitude lower osmolyte concentration than in the gallbladder.

## Data Availability

No datasets were generated or analysed during the current study.
